# The left axillary artery is a reasonable option as the inflow site for saphenous vein graft in minimally invasive coronary artery bypass grafting

**DOI:** 10.3389/fcvm.2024.1397396

**Published:** 2024-08-21

**Authors:** Ryohei Ushioda, Aina Hirofuji, Dit Yoongtong, Boonsap Sakboon, Jaroen Cheewinmethasiri, Thanin Lokeskrawee, Jayanton Patumanond, Suppachai Lawanaskol, Hiroyuki Kamiya, Nuttapon Arayawudhikul

**Affiliations:** ^1^ Cardiovascular and Thoracic Surgery Unit, Department of Surgery, Lampang Hospital, Lampang, Thailand; ^2^ Department of Cardiac Surgery, Asahikawa Medical University, Asahikawa, Japan; ^3^ Department of Emergency Medicine, Lampang Hospital, Lampang, Thailand; ^4^ Center for Clinical Epidemiology and Clinical Statistics, Faculty of Medicine, Chiang Mai University, Chiang Mai, Thailand; ^5^ Analysis Department, Chaiprakarn Hospital, Chiang Mai, Thailand

**Keywords:** off-pump coronary artery bypass grafting, left axillary artery, saphenous vein graft, minimally invasive off-pump coronary artery bypass grafting, subclavian/axillary artery to coronary artery bypass

## Abstract

**Introduction:**

This study aims to clarify the good inflow site for saphenous vein grafts (SVG) in minimally invasive off-pump coronary artery bypass grafting (mini-CABG), between the ascending aorta, the internal thoracic arteries (ITAs) and the left axillary artery (LAA).

**Methods:**

This retrospective study included 126 patients who underwent Mini-CABG at our center between January 2014 and July 2023. Patients were divided into three groups according to the SVG inflow site for patency comparison: Aorta group (*n* = 56), LAA group (*n* = 23), and ITA group (*n* = 47).

**Results:**

There were 84 males, with mean age of 65.9 ± 7.0 years. There were no significant differences in preoperative characteristics between groups. Mean operation times were 254.6 ± 72.2, 213.7 ± 57.6, and 253.0 ± 81.2 min, and the average numbers of distal anastomoses were 2.9 ± 0.9, 2.4 ± 0.7 and 2.9 ± 1.1 in the Aorta, ITA and LAA groups respectively. Days in intensive care, hospital stay, and major complications did not differ between the groups. Early patency of SVG did not significantly differ among groups: 93.0% in the Aorta group, 98.0% in the ITA group, and 100% in the LAA group. Mean follow-up period was 136.7 ± 295.7 days, and follow-up coronary CTA revealed 18 SVG occlusions (Aorta group *n* = 8, ITA group *n* = 5, LAA group *n* = 5). The Kaplan-Meier curve for SVG patency rates did not show any significant differences among the three groups.

**Conclusion:**

The ascending aorta, the ITAs, and the LAA serve as reliable inflow sites with similar results in mini-CABG.

## 1 Introduction

The saphenous vein graft (SVG) has been longstanding as a very popular graft material for coronary artery bypass grafting (CABG). Although the ascending aorta has been traditionally recommended as the first choice for inflow in cases without severe aortic calcification (1), this notion should be reevaluated for CABG through the left mini-thoracotomy (mini-CABG: minimally invasive off-pump coronary artery bypass grafting). In mini-CABG, the ascending aorta was the previously preferred inflow site at our institute. However, our current strategy for SVGs has shifted to utilizing the internal thoracic arteries (ITAs) as our primary choice for inflow, to implement our aorta no-touch technique, and if the stenosis is more than 90% in the target coronary vessels. In cases where the ITA is too thin, or stenosis of the coronary target is less critical, the left axillary artery (LAA) is our second choice. Although the ascending aorta was originally our primary choice, it is now considered a last resort in our center. Especially with our shift in inflow site preference, we set out to clarify which inflow site is best for mini-CABG, especially regarding the LAA where previous studies are still limited. A study on the use of the axillary artery as an inflow source for revascularization of the coronary artery was reported by Coulson and Bakhshay in 1997 (2). Here, the minimally (or less) invasive coronary revascularization procedure is dubbed the subclavian/axillary artery to coronary artery bypass (SAXCAB) (3). Case reports and reviews of SAXCAB have since been reported, but the short-term patency compared to other inflows in mini-CABG remains unclear. Previous studies of SAXCAB reported 1–2-year patency rates of 70%–100%, with most of these reported cases being a single bypass to the left anterior descending artery (LAD) (3, 4), while SAXCAB is used for other various coronary arteries as a second conduit in our institute. We performed this retrospective study to investigate which is the good inflow site for SVG in mini-CABG, between the ascending aorta, the ITAs, and the LAA.

## 2 Patients and methods

From January 2014 to July 2023, 292 patients underwent mini-CABG in our center, out of which SVG was used in 148 patients, and a postoperative coronary computed tomography angiography (CTA) was done on 131 (88.5%) patients. Two cases of median sternotomy conversion and three cases with differing SVG inflow sites were excluded. Ultimately, 126 patients were included in this retrospective study. The patients were divided into three groups according to the inflow site of SVGs: the Aorta group (*n* = 56), the LAA group (*n* = 23), and the ITA group (*n* = 47). Before discharge, 136 SVG segments were evaluated, and early-term primary graft patency rates were compared between the three groups. Finally, during the follow-up period, 143 SVG segments were assessed, and these findings were utilized to obtain the mid-term patency rates of SVG through Kaplan-Meier curve analysis, and the risk factors for mid-term occlusion of SVGs were identified. A graft was defined as patent if not occluded on follow-up angiography. The institutional review board of Lampang Hospital approved this retrospective study and waived the need for written patient consent.

### 2.1 Surgical technique

#### 2.1.1 Surgical approach

The procedure was performed through a small 8–10 cm left mini-thoracotomy. This incision usually provided sufficient access to the ITAs and the heart using specialized instruments. Distal anastomosis was made using 8-0 polypropylene for arterial grafts, and 7-0 polypropylene for vein grafts.

#### 2.1.2 Access to the axillary artery

A 6 cm longitudinal incision 2 cm below and parallel to the left clavicle was made to expose the axillary artery. For all SAXCAB, the LAA was selected for inflow. The graft was anastomosed to the LAA using 6-0 polypropylene sutures. Then, the SVG was tunneled through the second intercostal space into the thoracic cavity. Special care was taken to avoid twisting and kinking of the graft during this process.

#### 2.1.3 ITA composite anastomosis

For graft anastomosis between the SVG and the ITA, the Y-composite technique was utilized in 47 cases (88.6%), and the I-composite technique in 6 cases (11.4%). Continuous 8-0 polypropylene sutures were made for these anastomoses. To maintain field stability for anastomosis, we had used a tissue stabilizer (Octopus® tissue stabilizer, Medtronic, Minneapolis, MN, USA) placed within the chest.

#### 2.1.4 Proximal anastomosis to the ascending aorta

In all cases, preoperative CT scans were done to evaluate aneurysm and calcification in the ascending aorta. Systolic blood pressure was controlled and maintained below 90 mmHg. To bring the ascending aorta closer to the surgeon, the pericardium at the left-hand side of the aorta was strung up with a heavy silk suture outside the chest onto the skin, and 3–4 moistened 4 × 4 cm2 gauzes were placed between the superior vena cava and the aorta. A Lambert-Kay Aortic Clamp was applied through the main thoracotomy incision, and a conventional aortic punch was used to punch out the anastomotic site. The proximal anastomosis was done with a continuous 6-0 polypropylene suture. In cases where the main pulmonary artery was so prominent that it obscured exposure of the ascending aorta, we utilized the Octopus® tissue stabilizer (Medtronic) to improve the surgical field.

### 2.2 Graft inflow strategy

The ascending aorta was the previously preferred inflow site at our institution. However, our current strategy for SVGs has shifted towards using ITAs as our primary choice, because we have come to prioritize the aorta no-touch technique to decrease the risk of perioperative cerebrovascular events (5). Within this study, the right ITA was selected in 3 cases (6.8%) and the left ITA in 41 cases (93.2%) as the SVG inflow site. In cases where the ITA is too narrow (diameter <1.5 mm), or the degree of stenosis of the coronary target is less critical (70%), we turned to LAA as our next option for inflow site. Although the ascending aorta was originally our primary choice, it is now considered a last resort in our center. In cases of left ITA injury, if the proximal remainder of the left ITA is still in good condition, an I-composite with SVG extension was constructed; if not, we converted to median sternotomy to utilize the right ITA.

### 2.3 Follow-up

Information on all causes of death and cardiac complications during the follow-up period was researched from the Lampang Hospital data bank. All patients in this study were followed up 2 weeks, 1 month, and 6 months post operation at our outpatient clinic. If the follow-up was lost, we called the family or the patients to verify their current status, and were able to achieve a 100% follow-up rate. The mean of clinical follow-up term was 659.8 ± 54.2 days. All patients were administered aspirin or clopidogrel post operation. Coronary CTA was performed in almost every patient with creatinine levels below 1.5 ng/dl before discharge. Further follow-up CTA was only considered for complex bypass cases, or discharged patients who developed clinical symptoms indicative of postoperative cardiac ischemia. The median [mini-max] of coronary follow-up days was 5 [2–2,465] days.

### 2.4 Statistical analysis

In continuous variables, the difference among groups was evaluated using the one-way ANOVA test or Kruskal-Wallis test. For categorical variables, chi-squared or Fisher's test were used. Statistical significance was set at *p* < 0.05. The Bonferroni correction was employed for multiple comparisons. A univariable or multivariable Cox regression analysis was used to identify independent risk factors of SVG occlusion, which was presented as the hazard ratio (HR) with 95% Confidence interval (CI). Any risk factors or prognostic factors with a *P* value <0.1 in the univariable analysis and other potential clinical confounders associated with it were included in the multivariable analysis. We tested for multicollinearity of independent factors before performing multivariable analysis. Primary graft patency rates were estimated using the Kaplan-Meier method, and compared using the log-rank test. The STATA Software/MP, Version 17.0 (Stata Corporation, College Station, Texas, USA), was used for the statistical analyses.

## 3 Results

### 3.1 Overall surgical results

Preoperative patient characteristics are listed on Table 1. In 126 patients, the mean age was 65.9 ± 7.0 years, with 84 males (66.7%). Percentages of patients with chronic renal disease (Aorta group 3.6%, ITA group 19.1%, LAA group 4.3%; *p* = 0.014) and peripheral arterial disease (PAD) (Aorta group 5.4%, ITA group 19.1%, LAA group 4.3%; *p* = 0.042) were significantly different between the three groups. Intraoperative results and short-term outcomes are on Table 2. There were significant differences in operating time (Aorta group 254.6 ± 72.2 min, ITA group 213.7 ± 57.6 min, LAA group 253.0 ± 81.2 min; *p* = 0.008), total number of grafts (Aorta group 2.5 ± 0.6, ITA group 2.2 ± 0.4, LAA group 2.2 ± 0.6; *p* = 0.015), and average number of distal anastomoses (Aorta group 2.9 ± 0.9, ITA group 2.4 ± 0.7, LAA group 2.9 ± 1.1; *p* = 0.014) between the three groups. The aorta no touch technique was implemented in all cases in the ITA group, most cases in the LAA group (Aorta group 0%, ITA group 100%, LAA group 91.3%; *p* < 0.001). There were no cases that required intraoperative IABP nor on-pump conversion. There were no significant differences in hospital stay, intensive care unit (ICU) stay, nor postoperative major complications. There were no surgical sight infection cases. One patient in the ITA group died within 30 days (0.8%). MACCE (major adverse cardiac or cerebrovascular event) occurred in 8 cases total (Aorta group 1.8%, ITA group 10.6%, LAA group 8.7%; *p* = 0.163) during postoperative follow-up. Cardiac death was observed in three cases, all in the ITA group. Postoperative myocardial infarction was reported in a total of three cases, one in each group; Out of these three cases, revascularization procedure was provided for one case in the Aorta group. Postoperative new stroke during follow-up was found in a total of two patients, one from the ITA group and one from the LAA group.

**Table 1:** Patient characteristics and preoperative data (*n* = 126).

Variables	Aorta group (*n* = 56)	ITA group (*n* = 47)	LAA group (*n* = 23)	*P*-value
Age, mean ± SD years	65.0 ± 8.1	65.5 ± 8.0	68.9 ± 8.1	0.140
Male gender, *n* (%)	34 (60.7)	32 (68.1)	18 (78.3)	0.312
BMI, mean ± SD kg/m^2^	22.2 ± 3.3	22.6 ± 3.9	22.7 ± 4.1	0.807
NYHA class (≧Ⅲ), *n* (%)	10 (17.9)	12 (25.5)	1 (4.3)	0.098
Euro SCORE, median [IQR]	1.3 [1.0–2.6]	1.8 [1.1–3.4]	1.7 [1.0–2.7]	0.287
STS SCORE, median [IQR]	1.6 [0.8–2.5]	1.6 [0.9–3.3]	1.7 [1.1–3.4]	0.697
LVEF, mean ± SD %	57.8 ± 12.3	53.5 ± 13.9	56.7 ± 14.6	0.259
Comorbidity, *n* (%)				
Hyperlipidemia	52 (92.9)	47 (100)	23 (100)	0.076
Hypertension	54 (96.4)	47 (100)	23 (100)	0.281
Diabetes mellitus	25 (44.6)	28 (59.6)	8 (34.8)	0.112
Chronic renal disease (Cr ≧1.5 ng/dl)	2 (3.6)	9 (19.1)	1 (4.3)	0.014
Dialysis	1 (1.8)	6 (12.8)	1 (4.3)	0.068
COPD	3 (5.4)	5 (10.6)	3 (13.0)	0.460
Cerebral vascular accident	5 (8.9)	4 (8.5)	1 (4.3)	0.778
Peripheral arterial disease	3 (5.4)	9 (19.1)	1 (4.3)	0.042
STEMI	7 (12.5)	9 (19.1)	6 (26.1)	0.327
Recent myocardial infarction	29 (51.8)	27 (57.4)	14 (60.9)	0.721
Left main trunk lesions	29 (51.8)	19 (40.4)	7 (30.4)	0.188
Single vessel disease	2 (3.6)	2 (4.3)	0 (0)	0.618
Double vessel disease	14 (25.0)	21 (44.7)	7 (30.4)	0.102
Triple vessel disease	40 (71.4)	24 (51.1)	16 (69.6)	0.081
Preoperative PCI	4 (7.1)	7 (14.9)	5 (21.7)	0.177
Preoperative IABP	2 (3.6)	2 (4.3)	1 (4.3)	0.979
Urgency, *n* (%)				
Elective	50 (89.3)	36 (76.6)	17 (73.9)	0.141
Urgent	7 (12.5)	10 (21.3)	6 (26.1)	0.290
Emergent	0 (0)	0 (0)	0 (0)	
Salvage	0 (0)	1 (2.1)	0 (0)	0.429

BMI, body mass index; NYHA, New York Heart Association; STS, Society of Thoracic Surgeons; Euro SCORE, European System for Cardiac Operative Risk Evaluation; LVEF, left ventricular ejection fraction; COPD, chronic obstructive pulmonary disease; STEMI, ST-elevation myocardial infarction; PCI, percutaneous coronary intervention; IABP, intra-aortic balloon pumping.

**Table 2:** Operative data and postoperative short-term outcomes (*n* = 126).

Variables	Aorta group (*n* = 56)	ITA group (*n* = 47)	LAA group (*n* = 23)	*p*-value
Operative data				
Operating time, mean ± SD min	254.6 ± 72.2	213.7 ± 57.6	253 ± 81.2	0.008
Total grafts average, mean ± SD	2.5 ± 0.6	2.2 ± 0.4	2.2 ± 0.6	0.015
The average number of distal anastomoses, mean ± SD	2.9 ± 0.9	2.4 ± 0.7	2.9 ± 1.1	0.014
None touch aorta, *n* (%)	0 (0)	47 (100)	21 (91.3)	<0.001
Complete revascularization, *n* (%)	54 (96.4)	42 (89.4)	21 (91.3)	0.363
Over two atrial graft, *n* (%)	8 (14.3)	7 (14.9)	3 (13.0)	0.979
Graft, *n* (%)				
LITA	53 (94.6)	47 (100)	22 (95.7)	0.285
RITA	3 (5.4)	6 (12.8)	2 (8.7)	0.415
Radial artery	6 (10.7)	0 (0)	2 (8.7)	0.075
Postoperative outcome				
ICU stay, median [IQR] days	2 [1–2]	1 [1–2]	2 [1–2]	0.350
Hospital stay, median [IQR] days	6 [5–7]	6 [5–7]	5 [5–6]	0.727
Extubation within 24 h, *n* (%)	52 (92.9)	44 (93.6)	22 (95.7)	0.898
Perioperative transfusion, *n* (%)	36 (64.3)	24 (51.1)	13 (56.5)	0.395
Drain contents, median [IQR] ml	350 [300–550]	340 [240–500]	460 [280–600]	0.240
30-day mortality, *n* (%)	0 (0)	1 (2.1)	0 (0)	0.429
Postoperative complications, *n* (%)				
New stroke	0 (0)	1 (2.1)	0 (0)	0.429
New dialysis	0 (0)	0 (0)	0 (0)	
New onset atrial fibrillation	14 (25.0)	7 (14.9)	9 (39.1)	0.079
Wound infection	0 (0)	0 (0)	0 (0)	
Reoperation of bleeding	2 (3.6)	1 (2.1)	1 (4.3)	0.861
MACCE long-term, *n* (%)	1 (1.8)	5 (10.6)	2 (8.7)	0.163
Cardiac death	0 (0)	3 (4.3)	0 (0)	0.076
Stroke	0 (0)	1 (2.1)	1 (4.3)	0.348
Hear failure requiring hospitalization	0 (0)	0 (0)	0 (0)	
Postoperative myocardial infarction	1 (1.8)	1 (2.1)	1 (4.3)	0.786
Repeat revascularization	1 (1.8)	0 (0)	0 (0)	0.533

LITA, left internal thoracic artery; RITA, right internal thoracic artery; ICU, intensive care unit; MACCE, major adverse cardiac or cerebrovascular events.

### 3.2 Details of used SVGs

**Figure 1:**
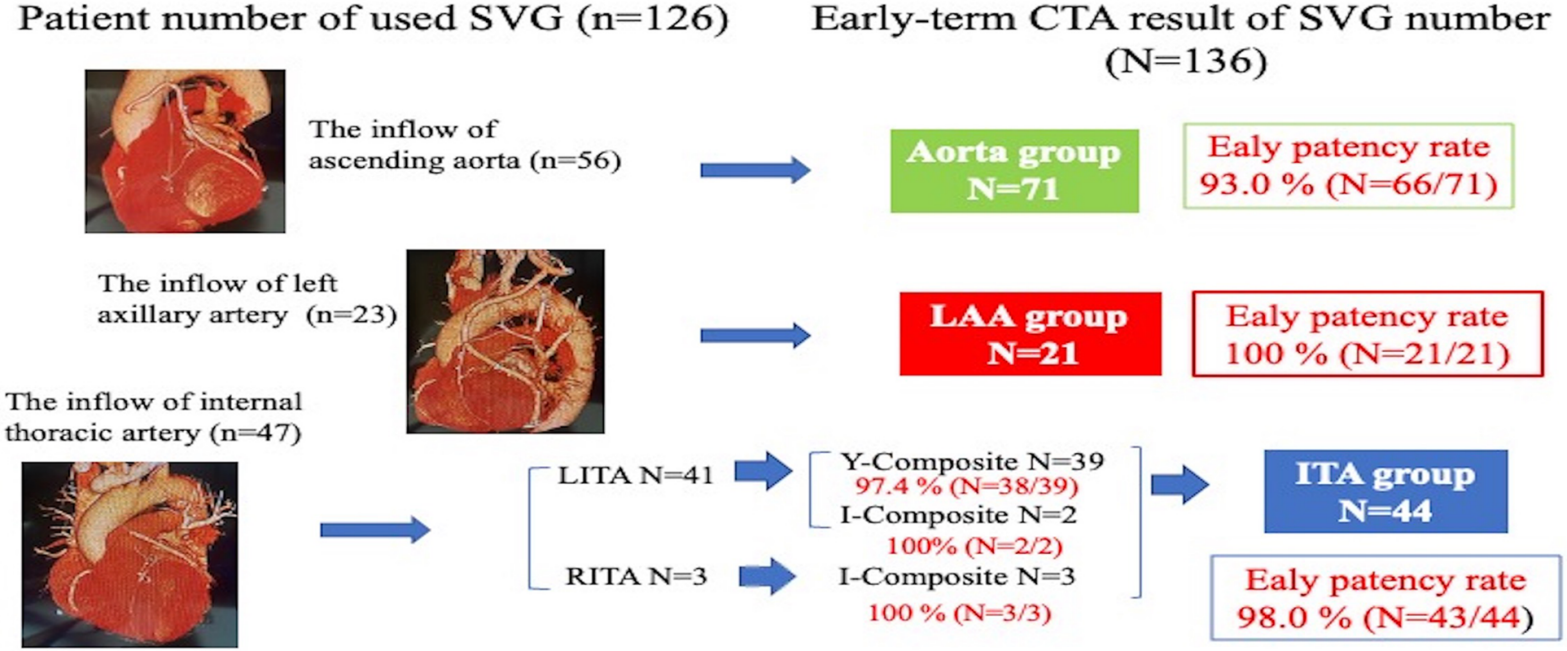
Early-term coronary computed tomography angiography result of saphenous vein graft patency (*p* = 0.271).

The SVG details according to inflow sites are organized on Table 3 (*n* = 136). There was a significant difference in the number of sequential technique (Aorta group 25.4%, ITA group 38.6%, LAA group 61.9%; *p* = 0.007). Significant differences between the three groups were seen in distal anastomosis in the left circumflex artery/branches (Aorta group 52.1%, ITA group 77.3%, LAA group 76.2%; *p* = 0.011) and the right coronary artery/branches (Aorta group 53.5%, ITA group 29.5%, LAA group 61.9%; *p* = 0.015). Early patency of SVG did not differ significantly among groups: 93.0% in the Aorta, 98.0% in the ITA, and 100% in the LAA groups (*p* = 0.271) (Figure 1).

**Table 3:** Details of saphenous vein graft evaluated in early-term CTA (*n* = 136).

	Ascending aorta (*n* = 71)	Internal thoracic artery (*n* = 44)	Left axillary artery (*n* = 21)	*p*-value
Number of distal anastomoses with vein graft, mean ± SD	1.31 ± 0.60	1.41 ± 0.54	1.62 ± 0.59	0.101
Sequential technique, *n* (%)	18 (25.4)	17 (38.6)	13 (61.9)	0.007
Early-term saphenous vein graft patency rate, *n* (%)	66 (93)	43 (98)	21 (100)	0.271
Distal anastomoses area, *n* (%)				
Left anterior descending artery	3 (4.2)	2 (4.5)	1 (4.8)	0.993
Diagonal branch	9 (12.7)	8 (18.2)	3 (14.3)	0.719
Circumflex territory	37 (52.1)	87 (77.3)	16 (76.2)	0.011
Right coronary system	38 (53.5)	13 (29.5)	13 (61.9)	0.015

### 3.3 Mid-term patency

**Figure 2:**
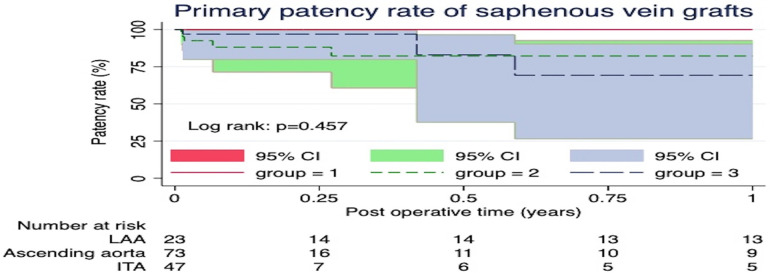
The Kaplan-Meier curve for saphenous vein graft patency rates showed no significant differences among the three groups (*p* = 0.457).

Postoperative follow-up coronary CTA revealed a total of 18 SVG occlusions (12.6%). The univariable analysis indicated that risk factors for mid-term occlusion of SVGs included the female gender, New York Heart Association class >3, PAD, and recent myocardial infarction (Table 4). However, utilization of the sequential technique, distal anastomoses sites, nor proximal anastomoses sites were not associated as SVG occlusion risk factors. Only the female gender was a risk factor for mid-term SVG occlusion in the multivariable analysis (HR = 3.59, 95% CI = 1.27–10.14, *p* = 0.016). The Kaplan-Meier curve for SVG patency rates showed no significant differences among the three groups (*p* = 0.457) (Figure 2). The one-year patency rates were 82.2% (*n* = 9) in the Aorta, 69.2% (*n* = 4) in the ITA, and 100% (*n* = 13) in the LAA (*p* = 0.138) groups.

**Table 4:** Univariable and multivariable analyses for factors associated with saphenous vein graft mid-term patency.

Variable	Univariable analysis			Multivariable analysis		
	HR	95% CI	*P* value	HR	95% CI	*P* value
Preoperative factor						
Age	1.03	0.97–1.10	0.319			
Female gender	3.20	1.18–8.69	0.022	3.59	1.27–10.14	0.016
BMI	0.99	0.86–1.14	0.905			
NYHA class (≧Ⅲ)	3.07	0.81–11.63	0.099	0.92	0.17–5.03	0.921
Diabetes mellitus	1.02	0.39–2.65	0.975			
COPDA	1.14	0.26–5.08	0.855			
Cerebral vascular accident	1.26	2.78–5.70	0.766			
Peripheral arterial disease	3.52	0.94–13.20	0.061	1.95	0.37–10.34	0.434
ST-elevation myocardial infarction	0.43	0.06–3.32	0.420			
Recent myocardial infarction	2.83	0.91–8.81	0.072	3.29	0.99–10.95	0.052
Left main trunk lesions	1.09	0.41–2.86	0.863			
Double vessel disease	1.82	0.70–4.73	0.222			
Triple vessel disease	0.83	0.32–2.17	0.699			
Preoperative PCI	0.58	0.08–4.50	0.604			
LVEF	0.97	0.21–44.88	0.986			
Elective	1.02	0.23–4.56	0.973			
Urgent	1.60	0.45–5.69	0.468			
Operative factor						
Number of distal anastomoses with vein graft	0.76	0.31–1.89	0.560			
Sequential technique	0.63	0.22–1.80	0.390			
Proximal anastomoses site						
Ascending aorta	1.15	0.43–3.11	0.776			
Internal thoracic artery	1.69	0.58–4.90	0.338			
Left axillary artery	2.29	0.73–7.19	0.157			
Distal anastomoses area						
Left anterior descending artery	0.76	0.09–6.04	0.791			
Circumflex territory	1.50	0.42–5.29	0.530			
Right coronary system	0.93	0.34–2.58	0.891			

BMI: body mass index; NYHA, New York Heart Association; COPD, chronic obstructive pulmonary disease; STEMI, ST-elevation myocardial infarction; PCI, percutaneous coronary intervention; IABP, intra-aortic balloon pumping; LVEF, left ventricular ejection fraction.

## 4 Discussion

### 4.1 SVG inflow from the ascending aorta and ITA

Since Favaloro et al. reported the first planned use of the SVG as an aortocoronary bypass graft in 1967 (6), the ascending aorta has steadfastly remained the preferred choice for SVG inflow. Meanwhile, the use of ITA as inflow for a composite SVG remains controversial. Recent studies show similar results between SVG and arterial composite grafts in terms of early and mid-term graft patency rates and clinical outcomes (7–9). A randomized trial comparing the SVG (SVG group, *n* = 112) and right ITA (right ITA group, *n* = 112) as Y-composite grafts anastomosed onto the *in situ* left ITA (9) concluded that the SVG composite grafts were equivalent to arterial composite grafts in terms of 5-year graft occlusion rate (3.5% in the SVG group vs. 3.7% in the right ITA group; *p* = 0.910). However, some previous reports demonstrated that SVG composite grafts anastomosed to *in situ* ITAs as a T- or Y- graft was suboptimal in terms of short-term patency when compared to arterial grafts, because SVG may cause more flow diversion than arterial grafts, resulting in reduced flow capacity on the ITA downstream of the composite site (10–12). In our institution, the ITA has been the preferred inflow choice for mini-CABG. Our retrospective analysis did not reveal significant differences in the Kaplan–Meier curve of mid-term SVG patency rates among the three inflow sites (ascending aorta, ITAs, and LAA). Long-term patency rates still remain unclear, and we will continue to follow up on this study.

### 4.2 Indication and exclusion criteria for SAXCAB

Some indications for SAXCAB include: ITA complications during minimally invasive CABG, untouchable ascending aorta, high risk reoperations, and severe chronic obstructive pulmonary disease. Most SAXCAB procedures reported in previous studies are reoperations (11, 12). Although our study didn't include reoperation cases, Minakawa et al. concluded that SAXCAB can be helpful in preventing injury to patent grafts caused by median sternotomy, and is also a useful strategy for reoperative off-pump CABG (12). Recently, several reports have described SAXCAB as a method to achieve complete revascularization in CABG with left mini-thoracotomy (13, 14). Lear et al. and Bonatti et al. reported using axilla-coronary bypass to provide totally endoscopic multivessel CABG (15, 16). We performed SAXCAB in cases where the ITA was too narrow (diameter of <1.5 mm), or the degree of stenosis of the coronary target was less critical (70%); in such cases, the LAA is our runner-up choice as the inflow site, not the ascending aorta, as there is no need for intraoperative evaluation of the aorta and its condition. Athanasiou et al. presented SAXCAB as a bailout procedure for a proximal left ITA injury using minimal access (17). In our study, two patients required SAXCAB for this very situation. The only exclusion criteria for SAXCAB are axillary artery disease (stenosis, aneurysm, infection, etc.), and dialysis shunt in the ipsilateral upper limb. Because the exclusion criteria is limited, SAXCAB is inclusive of most patients. Therefore, we did not routinely perform preoperative CTA to rule out these diseases. Another advantage is, because the LAA anastomoses field is easily accessible, the proximal anastomosis can be easily done.

### 4.3 SAXCAB grafts and its best routes

Various methods for tunneling SAXCAB grafts into the chest cavity have been reported; These include a subcutaneous route, a subfascial plane route, a tunnel through the second costal cartilage bed, and a tunnel through the intercostal muscles (3). Although the best route for SVGs is still debatable, recent reports showed that the graft inside the chest often travels through an incision at the first or second intercostal space. In similar fashion, we also made an incision in the first intercostal space to place the SVG into the thoracic cavity in our SAXCAB procedures. Some techniques to avoid mechanical trauma of the SVG, and strangulation of the graft route due to neointimal proliferation have been reported. Kawata et al. recommend partial resection of the first or second rib (18). Athanasiou et al. used a Dacron conduit as an external stent in the SVG entrance to the chest cavity through the intercostal space (3). In our institution, we made a large point of entry for the SVG in the first intercostal space, making sure the entry was wide enough for one index finger to fit through. Ohtsuka et al. suggest utilizing endoscopy to check the intrapleural course of the graft for kinking or distortion (19). To summarize, some key points for optimal SVG routing are: After sufficient tunneling in the infraclavicular region, prepare a wide intercostal opening for the graft to travel through, and strategically place the graft in the pleural space that avoids entrapment or compression by the lung.

### 4.4 Graft choice and inflow side for SAXCAB

Selection of axillary artery inflow is possible on both sides. Anil et al. reported five reoperation cases of the right axillary artery to right coronary artery (RCA) bypass through a small anterior thoracotomy and partial sternotomy (20). However, this bypass technique from the right axillary artery is limited to the RCA. In our study, the left axillary artery (LAA) was selected in all cases. The graft material mostly commonly chosen for SAXCAB is the SVG. The main reason being, the graft needs to be as long as possible; Bonatti et al. concluded that the mean length of SVG required for the LAA to LAD bypass is 18.9 ± 2.8 cm, and the right axillary artery to RCA bypass is 26.0 ± 2.6 cm (21). The radial artery is sometimes selected as a bypass conduit in SAXCAB, but its short length makes it only viable for LAD or diagonal branch (22, 23) bypasses. In our study, SVG was used in all SAXCAB cases. In cases where the target was LAD or left circumflex artery/branch, SVG was harvested from above the knee. For cases targeting RCA and or its branches, SVG was harvested in its entire length.

### 4.5 Long-term patency of SAXCAB

The long-term patency rate of SAXCAB has been thought to be inferior to that of conventional aorta-coronary SVG because, the length of vein graft required for SAXCAB is significantly longer than that of the conventional aorta-coronary bypass, and the graft crosses several anatomical structures such as the intercostal space, the pleural cavity, and the pericardium. Coulson et al. reported a review of axially to coronary bypass, with an estimated total international experience of about 100 cases until the year 2000, and the patency was between 70 and 100 percent, 1–2 years post operation (3). More than 20 years after this report, approximately 100 additional cases have been reported. The outcome of SAXCAB regarding patency rate in the last two decades seems to be similar to that before year 2000 reported by Coulson et al. However, there has been no large studies focused on long-term patency of SAXCAB. Bonatti et al. reported that 13 of their axillary-coronary grafts (93%) were patent at a mean follow-up of seven months (20). Comparing the present study to these previous results, when the LAA was used as the inflow site, the 1-year patency rate was similar, with a maximum patency day of 1,012 days and counting.

### 4.6 Is the left axillary artery beneficial as an inflow for CABG with left mini thoracotomy?

Surgical revascularization through left thoracic minimal incision has stepped up from a single bypass of left ITA-LAD to multiple bypasses (24). At our institute, we have strived for complete revascularization in patients with multi-vessel disease, by utilizing SVGs. The mean number of grafts in the entire group was 2.3 ± 0.6, with a mean number of distal anastomoses totaling 2.7 ± 0.9. Complete revascularization was successfully achieved in 117 cases (92.9%). Some articles report that the sequential technique and multiple grafts from a common inflow are associated with higher graft occlusion risk (25, 26). Mehta et al. investigated the use of multiple SVGs vs. single SVG in CABG surgery in 3,014 patients. The results showed that multiple SVGs were associated with a higher 1-year vein graft failure rate (adjusted odds ratio 1.24, 95% confidence interval 1.03 to 1.48) (26). In our study, the mean number of distal anastomoses (Aorta group 1.3 ± 0.6, ITA group 1.4 ± 0.5, LAA group 1.6 ± 0.6; *p* = 0.101) and number of sequential technique (Aorta group 25.4%, ITA group 38.6%, LAA group 61.9%; *p* = 0.007) were highest in the LAA group. Nevertheless, these factors did not affect significant mid-term occlusion rates of SVGs in the univariable analysis. In addition, the one-year patency rates were 82.2% (*n* = 9) in the Aorta group, 69.2% (*n* = 4) in the ITA group, and 100% (*n* = 13) in the LAA group (*p* = 0.138). Considering our results, the LAA may be a good reliable option in multi-bypass CABG with a left mini-thoracotomy.

### 4.7 Study limitations

This study was a retrospective, nonrandomized analysis from a single center. In addition, evaluating the graft patency rate remains underpowered because the sample size and follow-up duration were insufficient. Previous randomized data have reported that the patency rate of no touch SVG is significantly better than that of the traditionally harvested SVG (27, 28); although no touch SVG was used in some cases in this study, there were no records to reveal the accurate number of these cases. The Kaplan-Meier method may not be appropriate for describing graft patency rate; graft failure was recorded to have occurred at the time of angiography, but this does not reflect the precise time of postoperative occlusion. The Kaplan-Meier method may also overestimate graft failure. In addition, the total number of LAA inflow cases was smaller than desired for this analysis. Further studies with more angiographic follow-up cases are required to determine long-term patency of LAA inflow SVGs.

## 5 Conclusion

Our SVG inflow strategy for mini-CABG demonstrated acceptable short-time results in carefully selected patients. SVG early patency and one-year patency rates showed similar results between inflows from the ascending aorta, ITAs, and LAA. The ascending aorta, the ITAs, and the LAA serve as reliable inflow sites with similar results in Mini-CABG.

The ascending aorta, the internal thoracic arteries, and the left axillary artery serve as reliable inflow sites in minimally invasive off-pump coronary artery bypass grafting.

## Data Availability

The raw data supporting the conclusions of this article will be made available by the authors, without undue reservation.

## References

[1] Nakamura M Yaku H Ako J Arai H Asai T Chikamori T JCS/JSCVS 2018 guideline on revascularization of stable coronary artery disease. Circ J. (2022) 86(3):477–588. 10.1253/circj.CJ-20-1282 35095031

[2] Coulson AS Bakhshay S. Axillary-coronary bypass. Ann Thorac Surg. (1998) 65(1):304–5. 10.1016/s0003-4975(97)01276-9 9456156

[3] Coulson AS Glasgow EF Bonatti J. Minimally invasive subclavian/axillary artery to coronary artery bypass (SAXCAB): review and classification. Heart Surg Forum. (2001) 4(1):13–25. 11502492

[4] Athanasiou T Kapetanakis EI Rao C Salvador L Darzi A. Axillary artery to left anterior descending coronary artery bypass with an externally stented graft: a technical report. J Cardiothorac Surg. (2008) 3:6. 10.1186/1749-8090-3-6 18269756 PMC2267781

[5] Pawliszak W Kowalewski M Raffa GM Malvindi PG Kowalkowska ME Szwed KA Cerebrovascular events after no-touch off-pump coronary artery bypass grafting, conventional side-clamp off-pump coronary artery bypass, and proximal anastomotic devices: a meta-analysis. J Am Heart Assoc. (2016) 5(2):e002802. 10.1161/JAHA.115.002802 26892526 PMC4802438

[6] Favaloro RG. Saphenous vein autograft replacement of severe segmental coronary artery occlusion: operative technique. Ann Thorac Surg. (1968) 5(4):334–9. 10.1016/s0003-4975(10)66351-5 5647919

[7] Hwang HY Lee KH Han JW Kim KB. Equivalency of saphenous vein and arterial composite grafts: 5-year angiography and midterm clinical follow-up. Ann Thorac Surg. (2016) 102(2):580–8. 10.1016/j.athoracsur.2016.02.017 27209611

[8] Hwang HY Lee Y Sohn SH Choi JW Kim KB. Equivalent 10-year angiographic and long-term clinical outcomes with saphenous vein composite grafts and arterial composite grafts. J Thorac Cardiovasc Surg. (2021) 162(5):1535–1543.e4. 10.1016/j.jtcvs.2020.01.109 32418633

[9] Kim MS Hwang HY Kim JS Oh SJ Jang MJ Kim KB. Saphenous vein versus right internal thoracic artery as a Y-composite graft: five-year angiographic and clinical results of a randomized trial. J Thorac Cardiovasc Surg. (2018) 156(4):1424–1433.e1. 10.1016/j.jtcvs.2018.04.123 30257282

[10] Gaudino M Alessandrini F Pragliola C Luciani N Trani C Burzotta F Composite Y internal thoracic artery-saphenous vein grafts: short-term angiographic results and vasoreactive profile. J Thorac Cardiovasc Surg. (2004) 127(4):1139–44. 10.1016/j.jtcvs.2003.07.051 15052214

[11] Tarakji AM Sinclair MC. Should axillary artery to coronary artery bypass be part of the cardiac surgeon’s armamentarium? Eur J Cardiothorac Surg. (2007) 32(1):65–8. 10.1016/j.ejcts.2007.03.046 17500005

[12] Minakawa M Takahashi K Kondo N Hatakeyama M Kuga T Fukuda I. Left thoracotomy approach in reoperative off-pump coronary revascularization: bypass grafting from the left axillary artery or descending thoracic aorta. Jpn J Thorac Cardiovasc Surg. (2003) 51(11):582–7. 10.1007/BF02736697 14650587

[13] Kawasaki Y Ozewa T Suenaga E. A case of MICS-CABG using the axillary artery for the proximal inflow source. Jpn J Cardiovasc Surg. (2021) 50:73–7. 10.4326/jjcvs.50.73

[14] Gutti R Phadke AU Goli NR Radhakrishna VC Rajput N. Minimal access coronary artery bypass in high grade aortic atheroma utilising left axillary artery for proximal anastomosis. Indian J Thorac Cardiovasc Surg. (2017) 33:38–40. 10.10007/s12055-016-0463-0

[15] Lehr EJ Zimrin D Vesely M Odonkor P Griffith B Bonatti J. Axillary-coronary sequential vein graft for total endoscopic triple coronary artery bypass. Ann Thorac Surg. (2010) 90(5):e79–81. 10.1016/j.athoracsur.2010.08.051 20971229

[16] Bonatti J Wehman B de Biasi AR Jeudy J Griffith B Lehr EJ. Totally endoscopic quadruple coronary artery bypass grafting is feasible using robotic technology. Ann Thorac Surg. (2012) 93(5):e111–2. 10.1016/j.athoracsur.2011.11.049 22541230

[17] Athanasiou T Ashrafian H Harling L Casula RP. Bailouts to LIMA damage for avoiding conversion in minimal access coronary procedures. Ann Thorac Surg. (2016) 102(2):e173–6. 10.1016/j.athoracsur.2016.02.059 27449460

[18] Kawata T Abe T Ueda T Taniguchi S. Modification of repeat lateral minimally invasive direct coronary artery bypass; left axillary artery to circumflex artery bypass. Kyobu Geka. (2002) 55:811–3. 12174630

[19] Ohtsuka T Kubota H Motomura N Takamoto S. Thoracoscopy for minimally invasive axillo-coronary artery bypass. Eur J Cardiothorac Surg. (2001) 20(4):856–7. 10.1016/s1010-7940(01)00926-5 11574241

[20] Apaydin AZ Oguz E Posacioglu H Calkavur T Ayik F Turhan S Reoperative off-pump right subclavian artery to right coronary artery bypass grafting without full sternotomy. J Card Surg. (2011) 26(2):148–50. 10.1111/j.1540-8191.2011.01216.x 21395682

[21] Bonatti J Ladurner R Hangler H Katzgraber F. Anatomical studies concerning technical feasibility of minimally invasive axillocoronary bypass grafting. Eur J Cardiothorac Surg. (1998) 14(Suppl 1):S71–5. 10.1016/s1010-7940(98)00109-2 9814797

[22] Bonatti J Coulson AS Bakhshay SA Posch L Sloan TJ. The subclavian and axillary arteries as inflow vessels for coronary artery bypass grafts–combined experience from three cardiac surgery centers. Heart Surg Forum. (2000) 3(4):307–11. discussion 311-2. 11178292

[23] Magovern JA Hunter TJ Yoon PD. Clinical results with left axillary to left anterior descending coronary artery bypass. Ann Thorac Surg. (2001) 71(2):561–4. 10.1016/s0003-4975(00)02460-7 11235706

[24] Yang D Zhang K Li J Wei D Ma J Wang Y Ninety-seven cases of experiences with the left thoracotomy approach for off-pump conventional revascularization: a retrospective cohort study. J Thorac Dis. (2022) 14(10):3915–23. 10.21037/jtd-22-1162 36389332 PMC9641355

[25] Ouzounian M Hassan A Yip AM Buth KJ Baskett RJ Ali IS The impact of sequential grafting on clinical outcomes following coronary artery bypass grafting. Eur J Cardiothorac Surg. (2010) 38(5):579–84. 10.1016/j.ejcts.2010.03.008 20579898

[26] Mehta RH Ferguson TB Lopes RD Hafley GE Mack MJ Kouchoukos NT Saphenous vein grafts with multiple versus single distal targets in patients undergoing coronary artery bypass surgery: one-year graft failure and five-year outcomes from the project of ex-vivo vein graft engineering via transfection (PREVENT) IV trial. Circulation. (2011) 124(3):280–8. 10.1161/CIRCULATIONAHA.110.991299 21709060 PMC5144829

[27] Samano N Geijer H Liden M Fremes S Bodin L Souza D. The no-touch saphenous vein for coronary artery bypass grafting maintains a patency, after 16 years, comparable to the left internal thoracic artery: a randomized trial. J Thorac Cardiovasc Surg. (2015) 150(4):880–8. 10.1016/j.jtcvs.2015.07.027 26282605

[28] Dreifaldt M Mannion JD Geijer H Lidén M Bodin L Souza D. The no-touch saphenous vein is an excellent alternative conduit to the radial artery 8 years after coronary artery bypass grafting: a randomized trial. J Thorac Cardiovasc Surg. (2021) 161(2):624–30. 10.1016/j.jtcvs.2019.09.177 31831193

